# Impact of Process Parameters on the Quality Attributes of High-Dose Metformin Hydrochloride Film-Coated Tablets

**DOI:** 10.3390/pharmaceutics18101264

**Published:** 2026-10-06

**Authors:** Nguyen Tan Duc, Dang-Khoa Nguyen, Huynh Thanh Liet, Nguyen Huynh Thiet, Phan Nguyen Truong Thang, Phu Loc Lu, Ngoc Hanh Ly, Duyen Thi My Huynh

**Affiliations:** 1 Faculty of Pharmacy, Can Tho University of Medicine and Pharmacy, Can Tho 900000, Vietnam; thienthong1987@gmail.com (N.T.D.); 2453030160@student.ctump.edu.vn (P.L.L.); lnhanh@ctump.edu.vn (N.H.L.); 2 School of Pharmacy, University of Medicine and Pharmacy at Ho Chi Minh City, Ho Chi Minh City 700000, Vietnam; ndkhoa@ump.edu.vn; 3 DHG Pharmaceutical Joint-Stock Company, Can Tho 900000, Vietnam; huynhthanhliet@gmail.com (H.T.L.); nht220799@gmail.com (N.H.T.); 4 Institute of Drug Quality Control-Ho Chi Minh City (IDQC HCMC), Ho Chi Minh City 700000, Vietnam; pntruongthang@gmail.com

**Keywords:** film coating, high-dose tablets, manufacturing process parameters, metformin hydrochloride, tablet quality attributes, wet granulation

## Abstract

**Background/Objectives**: Formulating high-dose metformin hydrochloride film-coated tablets is challenging due to the poor flowability and compressibility inherent to high drug loading. To ensure process robustness and consistent product quality, understanding the impact of manufacturing variables is crucial. This study evaluated the effects of key process parameters on the quality attributes of 1000 mg metformin hydrochloride tablets to establish optimal processing conditions for reliable production. **Methods**: The manufacturing process, comprising wet granulation, compression, and film coating, was investigated to evaluate the influence of key process parameters on tablet quality attributes. Granules were characterized for bulk density, tapped density, and compressibility index. Core tablets were evaluated for physical appearance, weight variation, hardness, and dissolution at a 45 min sampling point across various compression forces and tableting speeds. Film-coating efficiency was assessed based on coating quality, dissolution behavior, and stability. Favorable manufacturing conditions were empirically identified through single-variable optimization and statistical analysis. **Results**: Wet granulation conducted at a chopper speed of 1400 rpm for 7 min yielded granules with the lowest compressibility index (11.88%), demonstrating significantly improved flowability. Compression at a force of 10 kN and a speed of 40 rpm produced core tablets with acceptable appearance, robust hardness (7.06–7.37 kP), and complete drug release (100%). Tablet hardness was governed by a strong interaction between compression force and tableting speed (partial η^2^ = 0.929, *p* < 0.001) rather than by either factor alone: at 20 kN, hardness collapsed sharply once tableting speed reached 30 rpm, coincident with mild capping, identifying this force–speed combination as a process-failure condition. For the coating process, maintaining a tablet bed surface temperature of 40 °C ensured optimal coating uniformity without compromising product quality. Dissolution profile comparison against a coated reference product in the primary dissolution medium (pH 6.8) yielded f2 similarity values above 50 for all eight core tablet conditions, and stability testing under accelerated/thermal-stress conditions (up to 65 °C) confirmed maintained dissolution, assay, and related-substance (1-cyanoguanidine) compliance for both the test and reference products. The optimized manufacturing conditions successfully produced film-coated tablets that conformed to all predetermined quality specifications. **Conclusions**: Manufacturing process parameters significantly influenced the quality attributes of high-dose metformin hydrochloride film-coated tablets. Proper selection and control of these processing conditions ensured consistent granule properties, optimal tablet quality, and enhanced manufacturing robustness.

## 1. Introduction

Metformin hydrochloride remains the gold-standard first-line therapy for managing type 2 diabetes mellitus due to its established efficacy, favorable safety profile, and extensive clinical utility [1,2]. To accommodate high-dose therapeutic regimens and enhance patient compliance, metformin hydrochloride is commonly formulated into high-strength film-coated oral dosage forms, notably 1000 mg tablets. Commercially, metformin hydrochloride is marketed in both immediate-release and extended-release formulations. Immediate-release products are available at strengths of up to 1000 mg per tablet, whereas extended-release formulations are typically limited to lower single-tablet strengths, reflecting the swelling/gel-matrix technology commonly employed for controlled release, which constrains the maximum drug load achievable in a single extended-release unit [3,4]. Numerous immediate-release 1000 mg film-coated tablets, from both originator and generic manufacturers, are marketed globally. This established commercial precedent confirms both the clinical relevance and the manufacturing feasibility of the 1000 mg immediate-release format investigated in the present study, while underscoring that its production remains compounded by the same poor flowability, limited compressibility, and elastic recovery challenges [5,6,7]. However, manufacturing high-dose metformin formulations poses substantial challenges due to the poor physical properties inherent to the drug substance, including poor flowability, limited compressibility, and high elastic recovery [8,9,10]. These unfavorable characteristics frequently impair interparticle bonding during compression, thereby increasing the risk of mechanical defects such as capping, lamination, insufficient mechanical strength, and compromised product uniformity.

To overcome these limitations, wet granulation is extensively employed to optimize powder flow properties and enhance granule cohesiveness prior to compression [11,12]. Nevertheless, final product quality is governed not only by granulation parameters but also by critical processing variables during subsequent downstream operations, including tablet compression and film coating. Variations in these operating conditions can profoundly affect granule microstructural characteristics, tablet mechanical strength, drug release kinetics, and overall manufacturing reproducibility. Consequently, a systematic understanding of the interplay between manufacturing process parameters and product quality attributes is vital for developing robust production lines, particularly for formulations characterized by high drug loading [13,14,15,16].

While previous literature has underscored the significance of process parameter control in solid dosage manufacturing, comprehensive data evaluating the cumulative influence of manufacturing conditions across multiple sequential processing stages for high-dose metformin hydrochloride tablets remain scarce. Investigating the process dynamics throughout the granulation, compression, and coating stages could provide valuable insights to optimize manufacturing efficiency and safeguard product quality consistency [17,18,19].

Although single-factor process studies on conventional-dose tablets are well documented in the pharmaceutical literature, systematic, full-chain evaluations spanning wet granulation, compression, and film coating for high-dose (≥1000 mg), poorly compressible active pharmaceutical ingredients such as metformin hydrochloride remain scarce [20,21]. In particular, the compounded manufacturing risk that arises when defects from sequential processing stages (granule flow, tablet capping, and coating integrity) accumulate at this drug loading has not previously been characterized in an integrated manner. This gap limits the availability of practical, evidence-based processing guidance for manufacturers developing and scaling up high-dose metformin hydrochloride film-coated tablets.

Therefore, this study aimed to evaluate the impact of manufacturing process parameters during wet granulation, tablet compression, and film coating on the quality attributes of high-dose metformin hydrochloride 1000 mg film-coated tablets, systematically characterizing the tested operating conditions to identify favorable, empirically supported processing conditions for stable and reproducible tablet production.

## 2. Materials and Methods

### 2.1. Materials

The active pharmaceutical ingredient, metformin hydrochloride (EP grade, Aarti Drugs Limited, Mumbai, India), and core tablet excipients—including polyvinylpyrrolidone K30 (USP grade, Ashland, Wilmington, DE, USA), sodium starch glycolate (USP grade, Yung Zip Chemical Ind. Co., Ltd., Taichung, Taiwan), colloidal silicon dioxide (BP grade, Cabot GmbH, Rheinfelden, Germany), and magnesium stearate (USP grade, Peter Greven Asia Sdn. Bhd., Prai, Malaysia)—were used as received. Coating polymers and analytical reagents comprised hydroxypropyl methylcellulose 6 mPa·s (USP grade, Lotte Fine Chemical Co., Ltd., Ulsan, Republic of Korea), macrogol 6000 (EP grade, Sasol Germany GmbH, Hamburg, Germany), titanium dioxide (USP/JP grade, Precheza a.s., Přerov, Czech Republic), talc (EP grade, Imerys Talc, Luzenac, France), as well as potassium dihydrogen phosphate and sodium hydroxide (EP grade, Merck KGaA, Darmstadt, Germany).

The complete qualitative and quantitative composition of the metformin hydrochloride core tablet and the coating dispersion is summarized in Table 1. The core tablet formulation consisted of metformin hydrochloride (93.633% *w*/*w*, 1000 mg/tablet) as the active pharmaceutical ingredient, polyvinylpyrrolidone K30 (3.745% *w*/*w*, 40 mg/tablet) as the binder, sodium starch glycolate (0.936% *w*/*w*, 10 mg/tablet) as the disintegrant, colloidal silicon dioxide (0.749% *w*/*w*, 8 mg/tablet) as the glidant, and magnesium stearate (0.936% *w*/*w*, 10 mg/tablet) as the lubricant, yielding a theoretical core tablet weight of 1068 mg. The coating dispersion was composed of hydroxypropyl methylcellulose 6 mPa·s (45.0% *w*/*w* of coating solids) as the film-forming polymer, macrogol 6000 (30.0% *w*/*w*) as the plasticizer, titanium dioxide (15.0% *w*/*w*) as the opacifier, and talc (10.0% *w*/*w*) as the anti-tacking agent, corresponding to a nominal coating solids content of 20 mg per tablet [22].

### 2.2. Study Design and Selection of Process Parameters

This study established a systematic evaluation framework to investigate the impact of critical unit operations—specifically high-shear wet granulation, tablet compression, and film coating—on the quality attributes of 1000 mg metformin hydrochloride film-coated tablets [23,24]. The selection of specific process parameters was guided by the inherent physicochemical challenges of the drug substance, notably its high dose, poor flowability, limited compressibility, and pronounced elastic recovery. Consequently, the experimental design targeted processing variables that directly modulate granule micromeritics, interparticle bonding, mechanical strength, and coating integrity [25]. Through this approach, the relationships between operating variables and finished product quality were characterized to define a reliable manufacturing window. The investigated manufacturing stages, process parameters, and evaluated quality attributes are summarized in Table 2.

For the high-shear wet granulation stage, chopper speed and wet massing time were selected as they directly govern granule nucleation, densification, and subsequent flow behavior. During tablet compression, the investigation focused on compression force and tableting speed, which dictate interparticle bonding, mechanical strength, weight uniformity, and dissolution profiles. For the film-coating stage, the pre-coating drying conditions and tablet bed surface temperature were evaluated because these thermal parameters modulate solvent evaporation rates, thereby critical to coating uniformity, aesthetic appearance, and product stability [24,26].

Each manufacturing parameter was evaluated individually within predefined operating ranges via a single-variable optimization approach. The resulting intermediate granules, core tablets, and finished film-coated tablets were systematically characterized to establish the optimal processing windows required for reliable, stable production.

Parameters not selected as primary variables in this single-factor study—binder concentration, granulating fluid addition rate, dry-mixing time, coating dispersion solid content, spray rate, and atomization pressure—were held constant across all experimental runs at values established from preliminary trials and consistent with the equipment’s standard operating range; their fixed values are reported in the corresponding methodology subsections (Section 2.3.1 and Section 2.5.1). Binder concentration (36.36% *w*/*w* polyvinylpyrrolidone K30 in the granulating fluid) and the fluid addition rate were fixed at levels that provided adequate wet-mass consistency without inducing over-wetting in this high-dose metformin hydrochloride blend; dry-mixing time was fixed to ensure homogeneous powder blending prior to fluid addition, independent of the subsequently varied chopper speed applied during wet massing. Coating dispersion solid content, spray rate, and atomization air pressure were fixed at values selected to maintain a stable, non-clogging spray pattern across all tested bed-temperature and pre-coating drying conditions.

### 2.3. Evaluation of Wet Granulation Parameters

#### 2.3.1. Granulation Process Parameters

To overcome the poor flowability and limited compressibility inherent to high-dose metformin hydrochloride, a single-variable optimization approach was applied to evaluate two key granulation parameters: chopper speed (1400–2800 rpm) and wet massing time (3, 5, and 7 min). While one parameter was varied within these predefined ranges, all other formulation and processing variables were maintained constant. The resulting intermediate granules were collected for subsequent micromeritic characterization.

Wet granulation was carried out in a high-shear mixer granulator (bowl capacity: 5 kg; T & T MACHINERY, Ho Chi Minh City, Vietnam), fitted with a 3-paddle impeller operated at a fixed speed of 200 rpm (equipment design). Prior to wet massing, the powder blend underwent a dry-mixing period of 5 min at a chopper speed of 2800 rpm, a value fixed independently of the chopper speed subsequently applied during the wet massing step (the studied variable). The granulating fluid was prepared by dissolving 80 g of polyvinylpyrrolidone K30 in 140 g of purified water (binder concentration 36.36% *w*/*w*), for a total fluid volume of 220 g (10.29% *w*/*w* relative to the dry powder blend), added over approximately 30 s prior to the wet massing step described above. Wet granules were dried and sized before blending in a cube blender (5 kg capacity, Trung Viet, Ho Chi Minh City, Vietnam).

#### 2.3.2. Granule Characterization

The physical and flow properties of the prepared granules were assessed via bulk density, tapped density, and Carr’s compressibility index (*CI*). Bulk density (*d_bulk_*) was determined by measuring the volume of a known mass of granules in a graduated cylinder without external compaction. Tapped density (*d_tapped_*) was recorded after mechanically tapping the cylinder until a constant volume was achieved [27,28]. The compressibility index (*CI*, %) was subsequently calculated using the following equation:

(1) $$\mathrm{CI}\;=\;\left(\frac{\mathrm{dtapped}\;-\mathrm{dbulk}}{\mathrm{dtapped}}\right)\times 100$$

The *CI* value served as the primary indicator of granule flowability and packing behavior, where lower values indicated superior flow characteristics. Granule properties obtained from different operating conditions were compared statistically to identify the optimal granulation window.

The intermediate granules from each batch were further characterized for particle size distribution, moisture content, and granulation process yield. Particle size distribution was determined by sieve analysis using a Robot Sifter (Model RPS-01, Seishin Enterprise Co., Ltd., Tokyo, Japan) fitted with a sieve stack of 850, 500, 355, 250, 180, and 106 µm, operated at a vibration level of 70 and a frequency of 65 Hz, with a pulse interval of 1 s and a total sieving time of 5 min. The median particle diameter (D_50_) and the fine-particle fraction (percentage of granules passing through the 106 µm sieve relative to the total granule mass) were recorded in duplicate (*n* = 2) for each batch.

Moisture content was determined in triplicate (*n* = 3) by loss on drying (LOD), expressed as percentage weight loss, immediately after the drying step of each batch [29]. Granulation process yield was calculated as the ratio of the actual granule mass recovered after processing to the theoretical granule mass charged into the granulator, expressed as a percentage.

### 2.4. Evaluation of Tablet Compression Parameters

#### 2.4.1. Compression Process Parameters

The optimized intermediate granules were subjected to compression on a rotary tablet press to investigate the impact of two critical tableting variables: compression force (10–50 kN) and tableting speed (8–40 rpm). A single-variable optimization design was utilized, wherein one parameter was systematically varied while the other was kept constant. The resulting core tablets were collected for subsequent physical and mechanical characterization.

Compression was performed on a rotary tablet press (maximum output: 19,200 tablets/h, Syntegon, Waiblingen, Germany) fitted with 4 stations; tooling with oblong shape 19.45 mm, biconvex, smooth on both sides. The target theoretical core tablet weight was 1068 mg, consistent with the formulation composition provided in Table 1. Post-compression evaluation was conducted using a Mettler Toledo analytical balance (Mettler-Toledo, Greifensee, Switzerland) and a hardness tester (SOTAX, Aesch, Switzerland).

#### 2.4.2. Core Tablet Characterization

The quality attributes of the generated core tablets were comprehensively evaluated based on the following pharmaceutical criteria. *Physical Appearance:* Tablets were visually inspected immediately post-compression to detect macro-defects such as capping, lamination, sticking, or picking. *Tablet Thickness:* The thickness of the core tablets was measured using a digital thickness tester (*n* = 10) immediately after compression, to assess dimensional consistency across the tested compression force and tableting speed conditions. *Friability:* Tablet friability was determined in accordance with USP ⟨1216⟩ Tablet Friability using a friabilator. A sample of tablets corresponding to approximately 6.5 g (or 10 tablets, whichever governed) was de-dusted, weighed, and subjected to 100 revolutions at 25 rpm; the tablets were then de-dusted again and reweighed. Friability was calculated as the percentage weight loss relative to the initial tablet weight. *In Vitro Dissolution Profile:* Dissolution testing was performed using a Teledyne Hanson dissolution system (Teledyne Hanson Research, Chatsworth, CA, USA), Apparatus 1 (basket apparatus), operated at 100 rpm in 1000 mL of phosphate buffer (pH 6.8) maintained at 37.0 ± 0.5 °C. Samples were withdrawn at 45 min and quantified by UV-Vis spectrophotometry (Shimadzu Corporation, Kyoto, Japan) at 232 nm, using six tablets (*n* = 6) per tested condition; the acceptance criterion applied was ≥70% (Q) drug release at 45 min, consistent with the dissolution requirement specified in the USP monograph for Metformin Hydrochloride Tablets. To further contextualize dissolution performance, a complete dissolution profile (5, 10, 15, and 45 min) was additionally obtained for the eight core tablet conditions (A1–A8) in the primary dissolution medium (phosphate buffer, pH 6.8, as described above) and compared against a coated reference (innovator) product using the f2 similarity factor, calculated according to standard regulatory guidance: f2 = 50 × log{[1 + (1/n)Σ(Rt − Tt)^2^]^−0.5^ × 100}, where Rt and Tt are the mean percentage dissolved of the reference and test products, respectively, at each sampling time point, and n is the number of time points compared [30].

### 2.5. Evaluation of Film-Coating Parameters

#### 2.5.1. Coating Process Parameters

The compressed core tablets were coated in a perforated coating pan to evaluate the impact of two critical operational variables: pre-coating drying and tablet bed surface temperature (30–50 °C). Pre-coating drying was investigated using two distinct approaches: immediate coating post-compression versus coating following an additional dedicated thermal drying step [31,32]. Utilizing a single-variable optimization framework, individual parameters were systematically varied while all other processing and environmental conditions were held constant. The resulting film-coated tablets were harvested for subsequent quality and stability characterizations.

Film coating was performed in a perforated coating pan (O’Hara Technologies Inc., Richmond Hill, ON, Canada) (pan capacity: 1 kg; batch size: 200 tablets) equipped with a single spray nozzle (nozzle orifice size: 0.5 mm). Coating dispersion was applied at a spray rate of 0.654 g/min, atomization air pressure of 0.85 bar, pattern air pressure of 1 bar, and a pan rotation speed of 5 rpm, targeting a final coating weight gain of 1.87% *w*/*w* [33]. “Pre-coating drying” refers to an additional thermal drying/holding step applied to the core tablet bed immediately prior to the initiation of spray application of the coating dispersion, distinct from the tablet bed surface temperature maintained during active spraying; tablets in the “without drying” group proceeded directly from bed pre-warming to spray coating without this dedicated holding step. The solid content of the coating dispersion was 10% *w*/*w* (aqueous basis), held constant across all tested conditions in this study.

#### 2.5.2. Film-Coated Tablet Characterization

The final film-coated tablets were comprehensively characterized to ensure compliance with pharmaceutical standards. *Visual Appearance and Coating Quality:* Tablets were macroscopically inspected to evaluate film uniformity and identify processing-related defects, including orange-peel effects, surface roughness, color variation, bridging, or twins/sticking. *In Vitro Dissolution Profile:* Dissolution testing was conducted to determine whether the applied film-coating layer or its processing temperatures exerted an inhibitory effect on drug release kinetics compared to the core tablets. Tablets were packaged in Alu/Alu blister prior to storage, consistent with the intended commercial packaging configuration. *Assay:* Drug content was determined by UV-Vis spectrophotometry at 232 nm against a USP Metformin Hydrochloride RS standard, following the assay procedure specified in the USP monograph for Metformin Hydrochloride Tablets, which requires metformin hydrochloride content of 95.0–105.0% of label claim. *Stability Evaluation:* Selected film-coated batches, manufactured under the optimized processing conditions, were evaluated for stability alongside a reference (innovator) product. Both the test formulation and the reference product were initially characterized under standard long-term storage conditions (30 °C/75% RH) and were subsequently subjected to accelerated/thermal-stress conditions of 40 °C, 50 °C, and 65 °C, all maintained under controlled relative humidity (75% ± 5% RH), with samples withdrawn and evaluated after 1 and 2 weeks of exposure [34,35]. Post-storage, the samples were re-evaluated for physical appearance, in vitro dissolution profile, assay, and the related substance 1-cyanoguanidine, quantified by HPLC (Shimadzu Corporation, Kyoto, Japan) (acceptance limit ≤ 0.02%) [36].

### 2.6. Statistical Analysis

Descriptive statistics, including mean values and standard deviations, were calculated using Microsoft Excel (Microsoft 365; Microsoft Corp., Redmond, WA, USA). To evaluate the statistical significance of the manufacturing parameters on granule micromeritics and tablet quality attributes, data were analyzed using IBM SPSS Statistics software (version 27; IBM Corp., Armonk, NY, USA). The causal relationships between independent processing variables and the measured dependent responses were quantified using the General Linear Model (GLM) [37]. Inter-group comparisons were performed to delineate significant operational effects, with a *p*-value of less than 0.05 (*p* < 0.05) defined as the threshold for statistical significance.

For the compression-hardness dataset, two independent batches were manufactured per tested condition, and hardness was measured on ten core tablets per batch (2 batches × 10 tablets); the batch means were used as the experimental units in the General Linear Model. 

## 3. Results

### 3.1. Effects of Wet Granulation Parameters on Granule Properties

#### 3.1.1. Influence of Chopper Speed and Wet Massing Time on Granule Micromeritics

**Figure 1:**
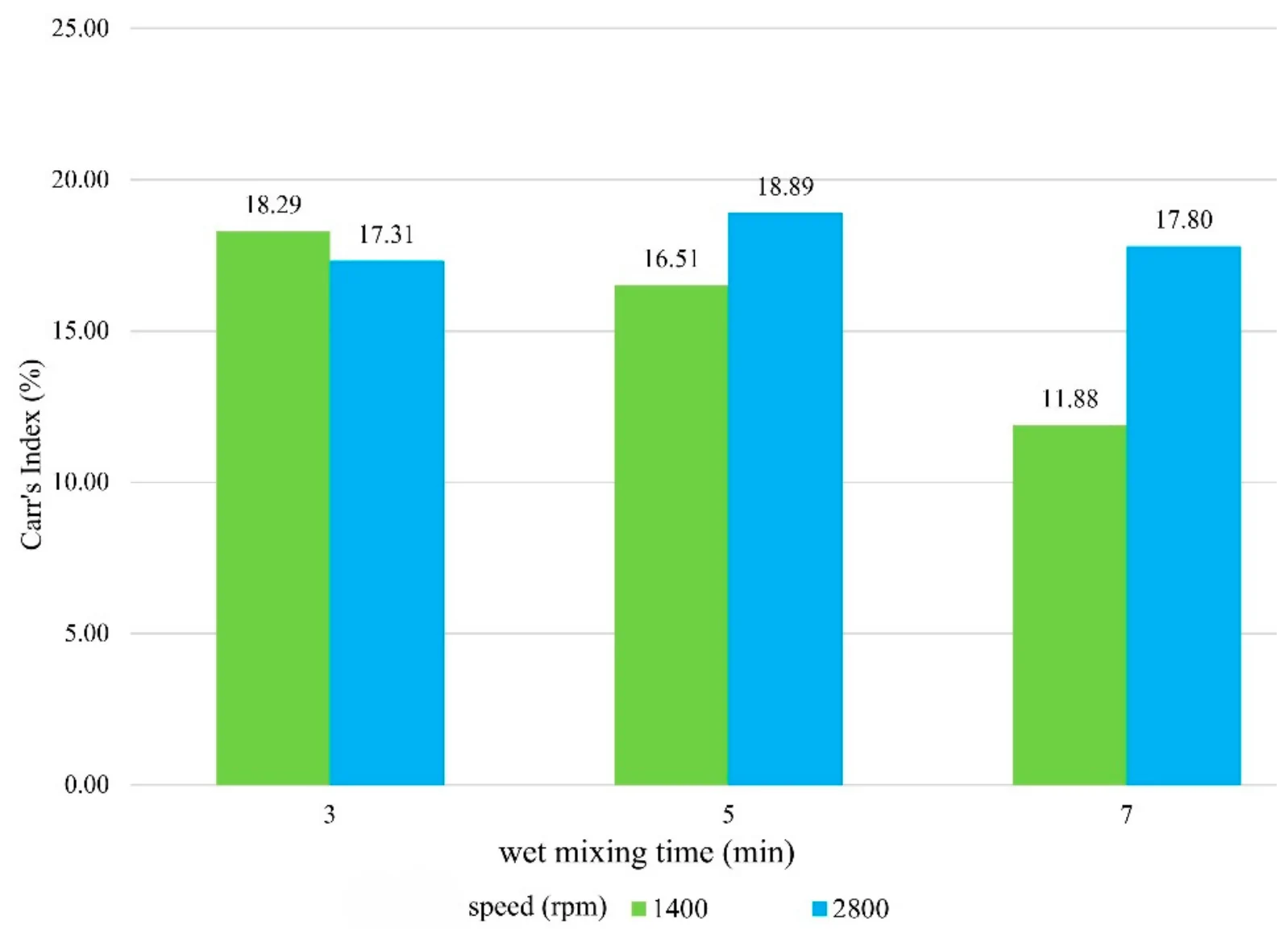
Effects of chopper speed and wet mixing time on granule compressibility index (CI).

The comprehensive effects of chopper speed and wet massing time on the micromeritic and flow properties of metformin hydrochloride granules are compiled in Table 3 and illustrated in Figure 1. The flowability and packing behavior of the intermediate granules were systematically quantified through the assessment of bulk density, tapped density, and the corresponding Carr’s compressibility index (*CI*).

At a constant chopper speed of 1400 rpm, extending the wet massing time from 3 to 7 min prompted a progressive and significant reduction in the compressibility index (*CI*) from 18.29% to 11.88%, signaling a substantial improvement in granule flowability. The lowest *CI* value (11.88%), reflecting superior flow characteristics, was achieved at the 7 min threshold. Concurrently, this prolonged wet massing induced a densification of the granule bed; the bulk density rose from 0.467 g/mL to 0.543 g/mL, while the tapped density increased from 0.572 g/mL to 0.616 g/mL.

In contrast, granules produced at 2800 rpm exhibited minimal variations in bulk density, tapped density, and compressibility index (*CI*: 17.31–18.89%) across all mixing times. This indicates that the impact of wet massing time is highly dependent on chopper speed. Under high-shear conditions (2800 rpm), granule consolidation appears to reach an immediate plateau. Consequently, a lower chopper speed (1400 rpm) combined with an extended wet massing time (7 min) proved superior, yielding the lowest *CI* (11.88%) and optimal flowability.

Particle size distribution, moisture content, and process yield of the intermediate granules under each tested granulation condition are summarized in Table 4.

At a constant chopper speed of 1400 rpm, extending the wet massing time from 3 to 7 min progressively increased the median granule size (D_50_: 257.75 → 508.94 µm) and reduced the fine-particle fraction (9.76% → 4.56%), consistent with the concurrent reduction in Carr’s Index (18.29% → 11.88%) reported in Section 3.1.1 and supporting progressive granule growth through liquid bridging and consolidation of fine particles into larger aggregates. At 2800 rpm, D_50_ and the fines fraction did not follow a monotonic trend with massing time (D_50_: 416.92, 392.73, and 495.45 µm at 3, 5, and 7 min, respectively; fines: 7.35%, 11.22%, and 7.16%), and the size distributions were markedly more variable (SD of D_50_ up to 134.09 µm), consistent with the irregular, shear-dominated consolidation proposed for high-chopper-speed conditions. Moisture content (LOD) remained tightly controlled across all batches (1.85–2.03%) regardless of chopper speed or massing time, indicating that the observed differences in Carr’s Index and particle size distribution were attributable to granulation dynamics rather than to residual moisture variation. Granulation process yield was consistently high across all conditions (95.55–96.85%) with no discernible trend, indicating that neither chopper speed nor wet massing time meaningfully affected process recovery within the tested ranges.

#### 3.1.2. Evaluation of Granule Compressibility Under Different Granulation Conditions

The granule compressibility index (*CI*), spanning from 11.88% to 18.89%, directly reflected the internal porosity and packing dynamics of the bulk powder. At 1400 rpm, lengthening the wet massing time from 3 to 7 min contracted the *CI* from 18.29% to 11.88%. This distinct reduction indicates that progressive liquid bridging and mechanical shear effectively consolidated the loose porous aggregates into denser, more spherical granules, thereby minimizing the volume reduction differential between the bulk and tapped states.

Conversely, operation at 2800 rpm yielded consistently high and stagnant *CI* values (17.31–18.89%) without a discernible kinetic trend. The excessive shear intensity at this higher speed likely induced rapid overwetting or irregular structural fracture, trapping air pockets within the granular framework and preventing optimal consolidation regardless of the time extension. Consequently, the 1400 rpm/7 min regime proved superior, establishing a well-consolidated granular state essential for uniform volume reduction during subsequent die filling.

### 3.2. Effects of Tablet Compression Parameters on Core Tablet Quality Attributes

#### 3.2.1. Influence of Compression Force and Tableting Speed on Tablet Physical Defects

The interactive effects of compression force and tableting speed on tablet macroscopic defects are summarized in Table 5. At a low compression force of 10 kN, tablets produced across the entire speed range (8–40 rpm) exhibited an intact, acceptable appearance without structural defects. Increasing the compression force to 20 kN maintained tablet integrity only at lower speeds (8–20 rpm); however, mild capping occurred as the tableting speed escalated to ≥30 rpm (Table 6, conditions A9–A10), consistent with the sharp hardness decline reported for these two conditions in Section 3.2.2.

At high compression forces (≥30 kN), mechanical defects became pervasive. Tablet capping was observed under the majority of investigated settings, while lower tableting speeds induced unstable machine operation due to excessive mechanical strain. These results indicate that high drug-loaded metformin formulations are highly sensitive to compression dynamics. Excessive compression forces combined with accelerated tableting speeds drastically amplify elastic recovery and internal stress concentration, leading to immediate structural failure (capping).

During process development, granule moisture content was identified as a critical factor influencing tablet defect formation: granules with moisture content below 1.6% produced tablets exhibiting capping/lamination-type surface delamination, whereas granules with moisture content above 2.4% resulted in punch sticking. Based on these preliminary observations, granule moisture content was therefore maintained within a narrower operating range of 1.7–2.3% for all compression batches reported in this study, although the effect of moisture level within this range was not systematically compared. Similarly, pre-compression force was held constant at 8.8 kN throughout the study to isolate and evaluate the effect of main compression force independently; the influence of varying pre-compression force itself was not investigated and represents a direction for future work.

#### 3.2.2. Influence of Compression Parameters on Tablet Weight Uniformity and Hardness

**Figure 2:**
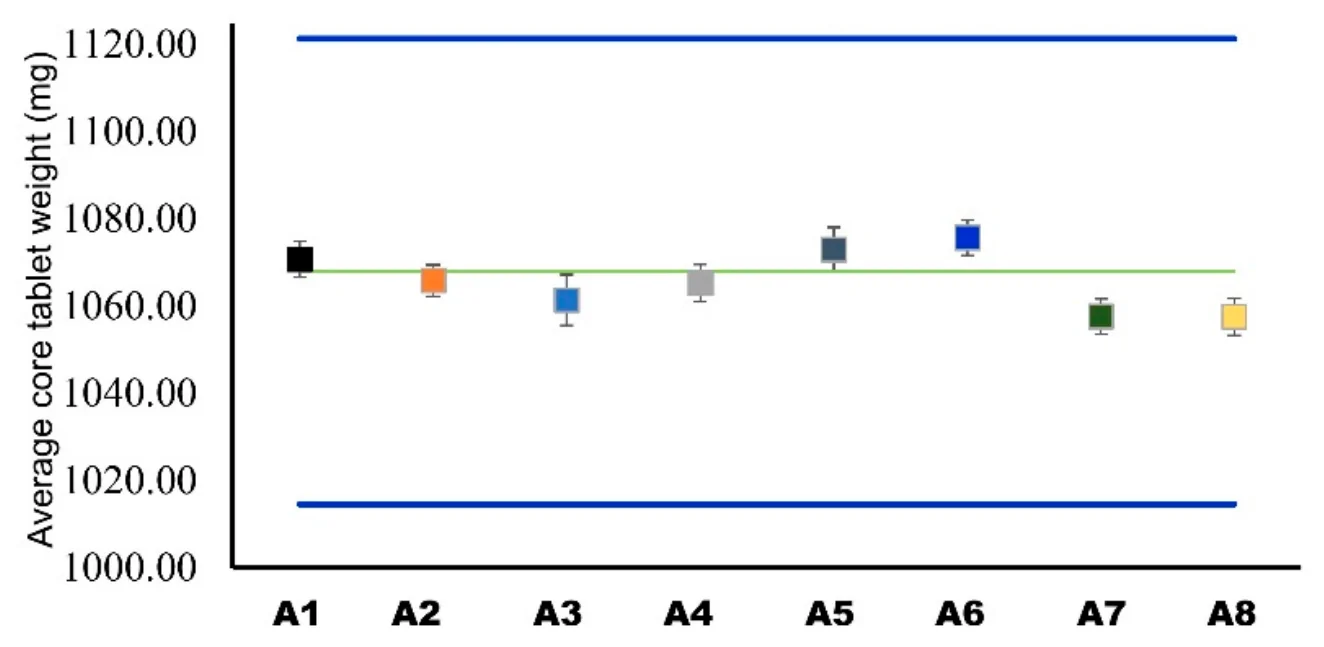
The effect of compression force and tableting speed on core tablet weight. The green line indicates the target core tablet weight (1068 mg), and the blue lines indicate the ±5% weight variation limits.

The average core tablet weight across all experimental batches ranged from 1057.59 to 1075.66 mg. The corresponding standard deviation (SD) values remained low, falling within a narrow interval of 3.58 to 5.81 mg, which demonstrates minimal intra-batch weight variation (Figure 2). Crucially, none of the evaluated batches contained individual tablets that deviated beyond the ±5% threshold from the calculated mean weight, thereby demonstrating strict compliance with the weight uniformity specifications stipulated by current pharmacopeial standards.

The resulting core tablet hardness values are summarized in Table 6. At 10 kN, hardness increased progressively with tableting speed, from 6.24 kP at 8 rpm to 7.22 kP at 40 rpm. At 20 kN, hardness remained high and essentially constant at 8 and 15 rpm (8.64 and 8.63 kP, respectively), declined slightly at 20 rpm (8.42 kP), and then collapsed sharply to 3.65 kP and 3.55 kP at 30 and 40 rpm, respectively (conditions A9–A10). This collapse coincided with the onset of mild capping at 20 kN/≥30 rpm (Section 3.2.1); tablets at these two conditions remained sufficiently intact for hardness testing, but the low recorded values reflect incipient structural weakening rather than fully intact compact strength.

Statistical evaluation via the General Linear Model (GLM) (Table 7), incorporating both experimental batches (2 independent batches × 10 tablets per condition across the 10 tested conditions), showed a statistically significant effect of tableting speed on tablet hardness (F(4,10) = 16.868, *p* < 0.001, partial η^2^ = 0.871).

The overall main effect of compression force was not statistically significant (F(1,10) = 0.110, *p* = 0.747, partial η^2^ = 0.011). However, a significant interaction between compression force and tableting speed was observed (F(4,10) = 32.806, *p* < 0.001, partial η^2^ = 0.929), indicating that the effect of compression force was entirely dependent on tableting speed (R^2^ = 0.952; adjusted R^2^ = 0.909). At 8–20 rpm, increasing the compression force from 10 to 20 kN increased tablet hardness. In contrast, at 30–40 rpm and 20 kN, mild tablet capping resulted in a marked reduction in measured hardness. These two high-force/high-speed combinations were therefore classified as process-failure conditions rather than acceptable operating conditions.

Tablet thickness (Table 6) provided corroborating physical evidence for this capping-driven collapse. At 10 kN, thickness declined modestly and consistently with increasing tableting speed (from approximately 7.9 mm at 8 rpm to 7.4–7.8 mm at 40 rpm), consistent with progressively more efficient consolidation. At 20 kN, thickness likewise decreased at 8–20 rpm (7.4–7.6 mm) but then increased sharply and became markedly more variable at 30–40 rpm (7.6–8.4 mm), coincident with the onset of mild capping. This anomalous thickness increase is consistent with the partial delamination characteristic of capping, in which localized separation of the tablet cap from the body increases the apparent tablet height despite the accompanying loss of measured hardness, and further corroborates the classification of the 20 kN/≥30 rpm combination as a process-failure condition.

#### 3.2.3. Influence of Compression Parameters on In Vitro Dissolution

**Figure 3:**
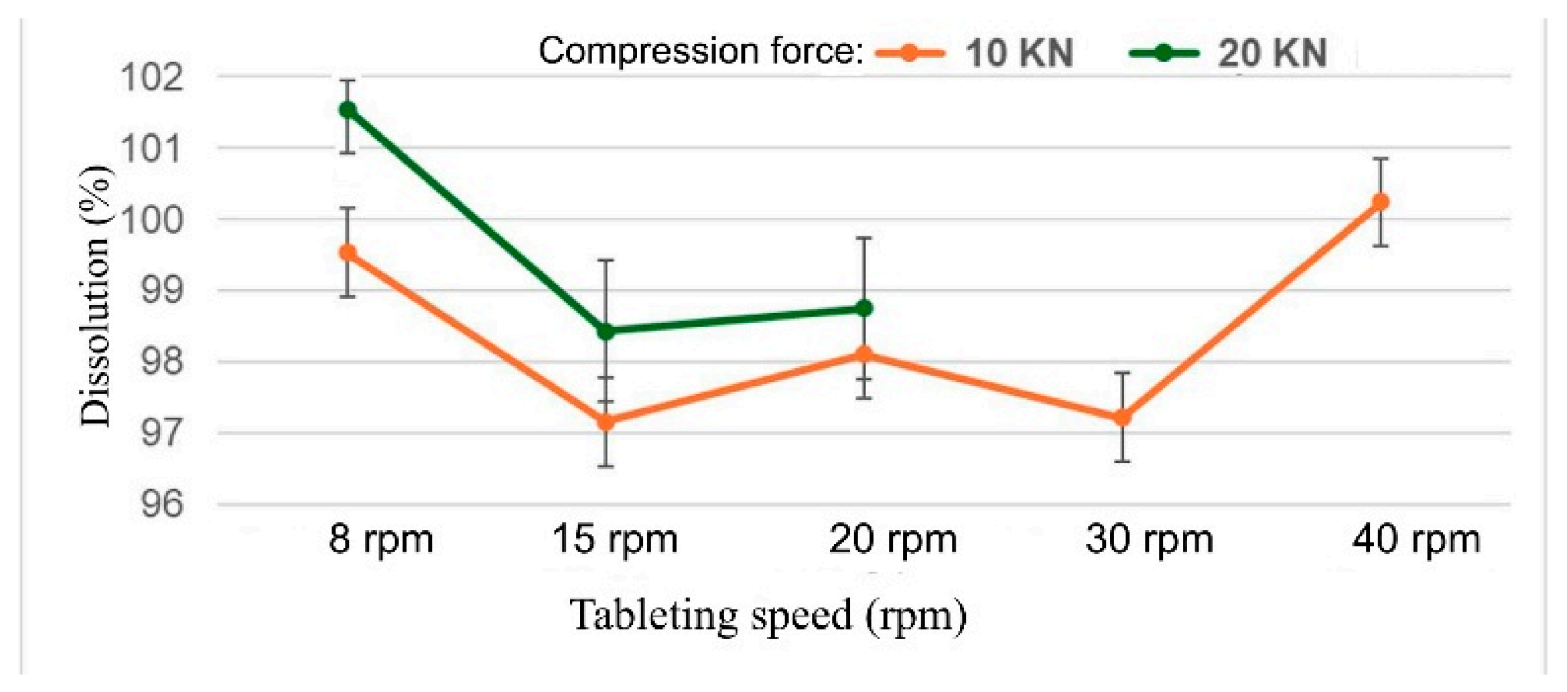
Effects of tableting speed and compression force on dissolution performance at 45 min (mean ± SD, *n* = 6).

The dissolution results of the core tablets under varying compression forces and tableting speeds at the 45 min sampling point are depicted in Figure 3, reporting the percentage of labeled drug content recovered at this single time point for each tested condition (mean ± SD, *n* = 6), rather than a complete multi-timepoint dissolution profile.

Across all investigated tableting configurations, the formulations achieved complete drug release at the 45 min checkpoint, with dissolution efficiencies hovering between 97.16% and 101.55%, consistently exceeding the predefined acceptance threshold.

Variations in compression force and tableting speed induced negligible differences in the drug release measured at the 45 min time point. Despite the distinct variations in core hardness and the occurrence of physical defects under certain extreme conditions (as detailed in Section 3.2.1 and Section 3.2.2), the 45 min release values remained highly uniform.

To further evaluate the dissolution behavior of the core tablets beyond the single 45 min endpoint, a complete dissolution profile was obtained for all eight compression conditions in the primary dissolution medium (phosphate buffer, pH 6.8) and compared against a coated reference (innovator) product using the f2 similarity factor (Table 8). All eight conditions yielded f2 values above the regulatory similarity threshold of 50, ranging from 52.91 (A5) to 76.20 (A2), indicating that the dissolution profile of the core tablets was broadly similar to that of the reference product across the tested compression conditions. Condition A5 (40 rpm/10 kN) showed the lowest similarity (f2 = 52.91), driven primarily by a markedly faster early-stage release (45.32% at 5 min) relative to the reference (33.62% at 5 min) and the other seven conditions (30.30–35.99% at 5 min); by 45 min, however, all conditions converged within the acceptance range described above. Because these comparisons were performed on uncoated core tablets against a coated reference product, the observed similarity should be interpreted as a preliminary, core-level benchmark rather than a formal product-to-product equivalence assessment; a corresponding comparison using the finished film-coated test tablets would be required to support a definitive bioequivalence-relevant conclusion.

To investigate the basis for this insensitivity, disintegration time was determined for the eight core tablet conditions not affected by capping (Table 9). Disintegration time ranged narrowly from 21.25 to 26.08 min and did not scale monotonically with hardness: for example, A6 and A7, among the hardest tablets tested (8.64 and 8.63 kP, respectively), disintegrated within 24.05–24.07 min—comparable to or faster than softer tablets such as A3 and A4 (26.08 and 26.05 min at 6.49 and 7.08 kP, respectively). This indicates that, within the tested range, core hardness was not the primary determinant of disintegration behavior. Because all tablets disintegrated within a comparatively narrow window (under 27 min) relative to the 45 min dissolution sampling point, sufficient time remained for the highly water-soluble metformin hydrochloride to fully dissolve regardless of the small differences in disintegration time between conditions. These disintegration data therefore substantiate, rather than merely presume, the proposed solubility-driven mechanism underlying the observed insensitivity of 45 min dissolution to compression force and tableting speed.

Friability was additionally assessed as a confirmatory (single-batch) measure of tablet mechanical robustness (Table 9). Friability values ranged from 0.41% to 0.59% across all eight conditions, remaining well within the ≤1% pharmacopeial acceptance limit regardless of compression force or tableting speed [38]; notably, friability decreased slightly at 20 kN relative to 10 kN (0.41–0.46% vs. 0.49–0.59%), consistent with the higher hardness observed at the higher compression force (Section 3.2.2, Table 6). As this friability assessment was performed on a single representative batch per condition, in contrast to the two independent batches used for the hardness and GLM analyses, it is reported here as descriptive, compendial-compliance evidence rather than as a statistically compared endpoint.

### 3.3. Effects of Film-Coating Parameters on Film-Coated Tablet Quality

#### 3.3.1. Influence of Pre-Coating Drying and Bed Temperature on Tablet Aesthetic Appearance

The structural and aesthetic impacts of pre-coating drying conditions and tablet bed surface temperatures on the finished film-coated tablets are summarized in Table 10. Across the entire experimental design space, all coated batches displayed an intact, uniform, and acceptable macroscopic appearance. Crucially, variations in tablet bed surface temperature (30–50 °C) and the integration of a dedicated pre-coating thermal drying step induced no visible processing-related defects. The film-coated matrices remained entirely free from typical coating abnormalities such as orange-peel effects, surface roughness, color variation, bridging, or sticking/picking. These findings indicate that the coating formulation possesses a robust processing window, yielding consistent film integrity and aesthetic quality within the evaluated operational boundaries.

#### 3.3.2. Influence of Film-Coating Conditions on In Vitro Dissolution

The dissolution efficiencies of the film-coated tablets under distinct processing configurations are compiled in Table 10. All evaluated batches achieved complete and rapid drug release at the 45 min interval, with final dissolution values tightly clustered between 98.81% and 102.40%, well above the predefined regulatory threshold.

The effects of pre-coating drying condition and tablet bed surface temperature on tablet appearance and dissolution performance are summarized in Table 10. All batches exhibited an acceptable film-coated tablet appearance across the tested bed-temperature range (30–50 °C), irrespective of the pre-coating drying condition.

To formally evaluate whether pre-coating drying condition and tablet bed surface temperature influenced dissolution performance, a two-factor ANOVA was performed at each sampling time point (Table 11). At 10, 15, and 45 min, neither tablet bed surface temperature nor pre-coating drying condition exerted a statistically significant effect on the percentage of drug released (all *p* > 0.14), confirming that the applied film-coating layer and its associated thermal processing conditions did not introduce a meaningful mass transfer barrier at these later time points. At the earliest sampling point (5 min), pre-coating drying condition showed a statistically significant effect (F = 15.584, *p* = 0.017), with batches subjected to pre-coating drying releasing, on average, 5.26 percentage points more drug than batches coated without this step (48.42% vs. 43.16%); tablet bed surface temperature had no significant effect at this time point (*p* = 0.529). This early-time difference is consistent with the inherently higher measurement variability typical of dissolution testing at 5 min, before the tablet matrix has fully disintegrated, and did not persist at any later time point: by 15 min, mean dissolution for both groups exceeded 96%, and by 45 min the two groups converged to within 0.3 percentage points of each other, both comfortably exceeding the predefined acceptance criterion. These findings indicate that pre-coating drying may modestly influence the very earliest phase of drug release, but does not affect the overall dissolution profile or the final extent of drug release; the film-coating process and its associated thermal exposure can therefore be considered compatible with the required dissolution performance of this high-dose metformin hydrochloride formulation across the tested range of bed-temperature and drying conditions.

### 3.4. Stability Evaluation of Film-Coated Tablets Under Thermal-Stress Conditions

#### 3.4.1. Alterations in Physical Properties During Thermal Stress Storage

The macroscopic and mechanical profiles of the film-coated tablets post-thermal stress exposure are summarized in Table 12. Across all thermal stress thresholds (40 °C, 50 °C, and 65 °C for 2 weeks), the tablets preserved an intact, acceptable appearance with no visible indications of coating damage, discoloration, cracking, or delamination. Furthermore, average tablet weight remained stable, with no individual core deviating beyond predefined weight variation tolerances.

In contrast to the static mass profiles, the mechanical strength of the tablets exhibited a distinct upward shift under thermal stress (Table 12). The developed formulation underwent a pronounced hardening effect, with crushing strength escalating from an initial 16.81 kP to a range of 24.73–31.97 kP following elevated temperature exposure. A comparable hardening kinetic was mirrored by the reference product. This phenomenon is frequently driven by the evaporation of residual moisture trapped within the tablet matrix or the thermal annealing of polymeric binders, which enhances solid-state interparticle bonding during stress storage.

The tablets subjected to thermal-stress testing in Table 12 originated from the same 10 kN/40 rpm compression batch reported in Table 6; no formulation or process change was introduced between the two stages. The substantially lower core hardness values in Table 6 (6.18–9.32 kP) reflect the granule moisture content deliberately maintained at approximately 2% during compression (Section 3.2.1) to prevent capping, a condition that limits interparticle bonding and yields a mechanically softer core tablet immediately after compression. Following film coating and subsequent exposure to thermal stress, however, progressive moisture loss from the tablet matrix—driven by the pre-coating drying/bed-temperature exposure during coating and further compounded by the elevated storage temperatures (40–65 °C) applied during thermal-stress testing—reduced the plasticizing effect of residual moisture and promoted additional solid-bridge formation between particles, consistent with the matrix-hardening/thermal-annealing mechanism proposed in Section 4. This combined effect accounts for the markedly higher hardness values observed in Table 12 (16.81–32.30 kP) relative to the freshly compressed core tablets in Table 6.

#### 3.4.2. Influence of Thermal Stress on In Vitro Dissolution

The dissolution efficiencies of the stress-tested formulations are compiled in Table 12. Post-exposure, the developed formulation maintained acceptable drug release kinetics at the 45 min interval, with dissolution values fluctuating within a compliant range of 88.90% to 101.35%. Although minor fluctuations in the dissolution profiles occurred across different thermal intensities and durations, all data points remained safely above the regulatory acceptance threshold.

The reference product exhibited a highly synchronous behavior, preserving its dissolution integrity under identical stress environments. Crucially, no progressive, temperature-dependent retardation of drug release was observed. These findings confirm that the structural hardening identified in Section 3.4.1 did not act as a rate-limiting barrier to mass transfer, ensuring that the critical in vitro performance of the high-dose metformin hydrochloride matrix remains robust against thermal fluctuations.

#### 3.4.3. Influence of Thermal Stress on Active Drug Content

The chemical stability of metformin hydrochloride under elevated thermal conditions was quantified via assay testing (Table 12). For the developed formulation, the active drug content remained exceptionally stable, tightly bound between 97.52% and 99.79% across all stress intervals. The minor variances in assay percentages represented typical analytical fluctuations rather than genuine chemical degradation, as no progressive downward trend in potency was identified with increasing temperature or prolonged storage.

This robust chemical recovery was fully aligned with the performance of the reference product, which likewise demonstrated strict compliance with drug content specifications throughout the study. Collectively, these results verify that the developed formulation possesses excellent solid-state chemical stability, capable of resisting thermal degradation under severe stress environments.

Related-substance analysis further confirmed the absence of thermally induced chemical degradation. The content of 1-cyanoguanidine, a known degradation product of metformin hydrochloride, remained essentially unchanged for the test formulation across all thermal-stress conditions (0.005–0.006%) relative to its initial value (0.006%), with no upward trend across increasing temperature or duration [39]. The reference product likewise maintained a low and stable 1-cyanoguanidine content (0.002–0.003%) throughout the study. Both formulations remained well within the acceptance limit of ≤0.02% under all tested conditions, corroborating the assay-based conclusion that the elevated temperatures and short-term exposure evaluated here did not induce meaningful chemical degradation of the active ingredient.

To facilitate comparison across the manufacturing stages and parameters investigated in this study, Table 13 summarizes the key process parameter–quality attribute relationships identified in the preceding results (Section 3.1, Section 3.2, Section 3.3 and Section 3.4).

## 4. Discussion

This study demonstrates that operational parameters across the wet granulation, tablet compression, and film-coating stages exert a systematic and profound influence on the critical quality attributes of high-dose (1000 mg) metformin hydrochloride film-coated tablets. Although this investigation adopted an empirical, single-variable optimization approach rather than a formal Quality by Design (QbD) framework, the findings consistently reveal strong, reproducible correlations between manufacturing conditions and product quality. Specifically, granule flowability, core tablet mechanical integrity, and coating performance proved highly sensitive to distinct operational windows. These results underscore the absolute necessity of rigorous process control when handling formulations characterized by an exceptionally high active pharmaceutical ingredient (API) load [40].

The manufacture of high-dose metformin hydrochloride is notoriously impeded by the drug’s poor intrinsic flowability and highly limited compressibility [18,41]. Consequently, the wet granulation phase serves as a vital gatekeeper for ensuring downstream manufacturability. In this work, the granule flow behavior—quantified via the Carr’s compressibility index (*CI*)—varied significantly as a function of chopper speed and wet massing duration [16]. The minimum *CI* value (11.88%) achieved under the 1400 rpm/7 min regime indicates the most favorable packing dynamics and flowability among the tested cohorts. This outcome aligns perfectly with classical wet granulation theory: adequate wet massing kinetics facilitate uniform binder distribution and promote progressive liquid bridging. This cohesive consolidation suppresses fines generation and enhances structural sphericity, ultimately yielding a granular bed with superior flow properties [42,43].

These particle size distribution (PSD) and moisture content results provide direct empirical support for part of the mechanistic interpretation proposed above. At 1400 rpm, the progressive increase in D_50_ and the concurrent decline in the fine-particle fraction with longer wet massing time mirror the reduction in Carr’s Index reported in Section 3.1.1, confirming that extended massing time promotes granule growth through liquid bridging and consolidation of fine particles rather than merely altering bulk packing behavior [15]. At 2800 rpm, the markedly higher variability in D_50_ across replicate measurements, together with the absence of a consistent trend with massing time, is consistent with an irregular, shear-dominated consolidation process that intermittently fractures and re-forms granule aggregates, yielding granules of inconsistent size despite comparable overall recovery. The tightly controlled moisture content across all batches (1.85–2.03%) indicates that the observed differences in *CI* and PSD are attributable to the mechanical dynamics of granulation rather than to residual-moisture variability, strengthening the internal validity of the chopper speed/massing time comparison. Likewise, the consistently high granulation yield (95.55–96.85%) across all tested conditions confirms that process losses did not differentially bias the granule properties compared between conditions [44]. Taken together, these findings reinforce the selection of the 1400 rpm/7 min condition as the granulation regime producing the most favorable downstream feed material—larger, less fine-laden granules.

At a broader level, the interplay between shear intensity and mixing duration governs the critical balance between granule growth and breakage. Lower chopper speeds coupled with sufficient wet massing durations favor controlled consolidation. Conversely, excessive mechanical energy input triggers granule attrition and heightened heterogeneity—a phenomenon widely documented in granulation science where aggressive processing disrupts granule size distribution and flowability [42]. In exceptionally high-dose formulations like metformin hydrochloride, maintaining this equilibrium is paramount; the extreme drug loading drastically restricts formulation flexibility and amplifies sensitivity to processing fluctuations. From a downstream manufacturing perspective, enhanced granule flowability optimizes die-filling dynamics during compression, thereby directly securing weight uniformity and tableting stability [16,18,41]. Thus, the wet granulation parameters optimized herein establish the fundamental framework required to ensure process robustness during subsequent compaction and coating operations.

Tablet compression parameters exerted a pronounced impact on the core tablets’ mechanical profiles and manufacturability. Compression force and tableting speed were identified as key variables governing hardness, appearance, and process stability; at 10 kN, elevating tableting speed from 8 to 40 rpm produced a modest, consistent increase in hardness (6.24–7.22 kP), consistent with classical compaction theory, wherein higher forces and residence times accelerate particle rearrangement, plastic deformation, and interparticulate bonding [45]. However, this relationship did not hold at the higher compression force: the GLM analysis (Section 3.2.2) revealed that compression force alone was not a statistically significant driver of hardness (partial η^2^ = 0.011, *p* = 0.747), and that hardness was instead governed by a dominant speed × force interaction (partial η^2^ = 0.929, *p* < 0.001) [46].

At 20 kN, hardness remained high (8.42–8.64 kP) up to 20 rpm, but then collapsed sharply at 30–40 rpm (Table 6, conditions A9–A10), coincident with the onset of mild capping (Section 3.2.1). Tableting speed on its own remained a significant, dominant main effect (partial η^2^ = 0.871, *p* < 0.001), but its influence was strongly conditional on force: at 10 kN it acted through the classical residence-time/die-filling mechanism described above, whereas at 20 kN, once speed reached the failure threshold, the same increase in speed instead precipitated structural collapse rather than continued hardening. This crossover pattern explains why compression force and tableting speed cannot be interpreted as independent drivers of hardness in this high-dose formulation, and identifies the combination of high force with high speed as the principal mechanical risk zone for this formulation [47].

Mechanistically, this crossover behavior can be rationalized by the competing roles of compression force and punch dwell time (governed by tableting speed) in particle bonding versus stress relaxation. At the lower force (10 kN), increasing speed shortens the dwell time but does not exceed the bonding capacity of the granule bed, so the modest reduction in interparticulate contact time is outweighed by more efficient die-filling and reduced air entrapment, yielding a small net increase in hardness; across all tableting speeds (8–40 rpm), this force maintained a defect-free appearance, defining a more stable operating window. At the higher force (20 kN), however, the same reduction in dwell time at high speed limits the time available for stress relaxation and plastic deformation immediately before ejection; the resulting elastic energy, insufficiently dissipated, is released abruptly upon die exit, manifesting as capping, consistent with highly non-uniform internal stress distribution within the compact compromising structural unity upon ejection [48,49,50]. This explains why tableting speed exerts opposite net effects on hardness depending on the force level, rather than acting as an independent, additive factor. Remarkably, despite these macro-structural defects and variations in hardness, the in vitro dissolution performance remained consistently high and uniform (97.2–101.6%) across all cohorts. This insensitivity is substantiated by the disintegration-time data (Section 3.2.3, Table 9), which showed no monotonic relationship with hardness across the eight non-capped conditions (21.25–26.08 min); because all tablets disintegrated well within the 45 min sampling window, the high intrinsic aqueous solubility of metformin hydrochloride was sufficient to drive complete dissolution regardless of the compression-induced differences in hardness and matrix porosity [51].This insensitivity is further corroborated by the complete dissolution profile comparison against a coated reference product in the primary dissolution medium (Section 3.2.3, Table 8): all eight compression conditions achieved f2 similarity values above the regulatory threshold of 50 (50.35–76.20), confirming that the compression-induced differences in hardness, thickness, and disintegration time observed across conditions did not translate into meaningfully different overall release kinetics relative to the reference product [52]. The single condition showing comparatively lower similarity (A5, 40 rpm/10 kN, f2 = 50.35) was driven by faster early-stage (5 min) release rather than by any deviation at the clinically relevant 45 min endpoint, further reinforcing that compression parameters within the tested range primarily affect the early dissolution phase rather than the extent of drug release.

Compared to the granulation and compaction stages, the film-coating process exhibited significantly lower sensitivity to operational variations. Within the investigated boundaries, both the integration of pre-coating drying and variations in tablet bed temperature (30–50 °C) consistently yielded film-coated tablets with excellent aesthetic appearance and rapid dissolution kinetics. The complete absence of visible coating defects—such as orange-peel effects, surface roughness, bridging, or sticking/picking—underscores the robustness of the coating formulation. Furthermore, the tightly clustered dissolution values (98.81%–102.40% at 45 min) confirmed that the applied polymeric coat introduced no mass transfer barrier. This conforms to the intended function of immediate-release film coats, which target aesthetic and stability enhancements without modifying drug release kinetics.

From a mechanistic standpoint, the tablet bed temperature is critical because it dictates solvent evaporation rates and film formation dynamics. Adequate thermal energy facilitates optimal droplet spreading and polymer particle coalescence, producing a continuous, uniform film [32,53,54]. Although suboptimal temperatures theoretically risk incomplete coalescence or prolonged drying, the investigated range (30–50 °C) remained safely within a stable operational window. Similarly, the negligible differences observed between tablets coated with or without a dedicated pre-coating drying step indicate that residual surface moisture was insufficient to impair film adhesion. This broad operational tolerance is highly advantageous for industrial scalability, as it minimizes final product variability against minor fluctuations in pre-coating handling.

The thermal-stress study confirmed that the developed film-coated tablets maintained overall structural integrity, mass uniformity, and chemical potency under accelerated conditions (40–65 °C for up to 2 weeks). Across all stress thresholds, no macro-defects—such as coating delamination, cracking, or discoloration—were detected, indicating excellent physical stability of the polymeric architecture. A notable finding was the pronounced post-storage hardening effect, with crushing strength escalating from an initial 16.81 kP under ambient conditions to 24.73–31.97 kP following thermal exposure. This kinetic was similarly mirrored by the reference product, demonstrating that the phenomenon is not formulation-specific but rather inherently linked to post-compression viscoelastic relaxation, late-stage consolidation of interparticulate solid bonds, or internal moisture redistribution during thermal incubation [55,56]. Importantly, this structural consolidation did not impair in vitro dissolution kinetics; both the test and reference formulations achieved compliant drug release (88.90–101.35%), exhibiting no temperature-dependent retardation trends. This confirms that mechanical tightening did not act as a rate-limiting barrier to mass transfer, a behavior directly sustained by the immediate-release nature of the matrix and the high water solubility of the active ingredient. Furthermore, chemical assay values remained tightly bound between 97.52% and 99.79% without a systematic degradation pattern, reinforcing the exceptional solid-state chemical stability of metformin hydrochloride under severe thermal regimes.

In an integrated manufacturing perspective, these findings suggest a hierarchy of process sensitivity for high-dose formulations, identifying the wet granulation and compression stages as the process steps warranting the closest control during scale-up [57,58]. Wet granulation directly acts as the primary gatekeeper for granule micromeritics, where the 1400 rpm/7 min regime yielded the minimum *CI* (11.88%) among the tested conditions, which is expected to support more consistent downstream die-filling, although die-filling behavior itself was not directly measured in this study. Meanwhile, the compression stage represents the critical control point for structural development, governed not by compression force alone but by a dominant speed × force interaction (partial η^2^ = 0.929, *p* < 0.001; Section 3.2.2), with the combination of high force and high speed constituting the principal mechanical risk zone; the tested condition of 10 kN/40 rpm represented a practical compromise among mechanical strength, defect-free appearance, and near-complete drug release, whereas 20 kN combined with tableting speeds of 30 rpm or higher should be regarded as a high-risk operating combination rather than a viable manufacturing condition. In contrast, the film-coating process and short-term thermal exposure showed lower sensitivity to the operational variations tested here. Maintaining stringent control over early unit operations therefore appears warranted to support reproducible granule properties and core tablet integrity, whereas the film-coating process tolerated the tested range of operating conditions with fewer visible or dissolution-related consequences [59].

This study has several limitations that should be acknowledged. First, the experimental design followed a single-variable (one-factor-at-a-time) approach rather than a formal multivariate Design of Experiments (DoE); while this allowed clear attribution of individual parameter effects, it cannot fully capture higher-order interactions beyond those directly tested, nor establish a formal Quality-by-Design (QbD) design space. Second, granule morphology (SEM) was not characterized, so the proposed liquid-bridging and shear-fracture mechanisms remain indirectly supported by particle-size and density data rather than directly visualized. Finally, all experiments were performed at pilot scale using a single set of equipment; inherent batch-to-batch variability and equipment-specific effects were not assessed and may influence the transferability of the identified favorable conditions to industrial-scale manufacturing. Production-scale granulators typically operate with different impeller/chopper geometries and higher batch volumes, which can alter shear intensity and granule growth kinetics relative to the 5 kg bench-scale equipment used here. Similarly, industrial rotary presses often operate at substantially higher outputs with different punch dwell-time profiles and pre-compression configurations, which may shift the force–speed interaction boundary identified in this study. For film coating, larger pan diameters and batch sizes affect spray-to-surface distances, drying kinetics, and bed mixing efficiency, potentially altering the coating uniformity observed here. Confirmatory scale-up studies, ideally within a formal DoE and QbD framework, are therefore recommended before directly applying the favorable conditions identified here to commercial manufacturing.

## 5. Conclusions

This study empirically examined the impact of manufacturing parameters across the wet granulation, compaction, and film-coating stages on the quality attributes of high-dose (1000 mg) metformin hydrochloride film-coated tablets, using a single-variable optimization approach. The findings suggest a sensitivity hierarchy among unit operations, with the wet granulation and compression phases appearing most influential for intermediate and final product quality within the tested ranges. In the wet granulation stage, the 1400 rpm/7 min condition minimized the granule compressibility index to 11.88% among the tested conditions, indicating favorable flowability for downstream processing. During subsequent compaction, core tablet hardness was governed not by compression force alone but by a strong interaction between compression force and tableting speed (partial η^2^ = 0.929, *p* < 0.001; Section 3.2.2): hardness increased consistently with speed at 10 kN, whereas at 20 kN it remained high only up to 20 rpm before collapsing sharply at 30–40 rpm, coincident with the onset of mild capping. The tested condition of 10 kN/40 rpm represented a practical compromise among mechanical integrity, defect-free appearance, and near-complete drug release, whereas 20 kN combined with tableting speeds of 30 rpm or higher constitutes a high-risk operating combination rather than a viable manufacturing condition; neither should be regarded as a formally established optimum.

In contrast, the film-coating process tolerated the tested range of tablet bed surface temperature (30–50 °C) without introducing aesthetic defects or apparent mass transfer barriers to dissolution, within the operating conditions evaluated. Thermal-stress testing further indicated that the physical and chemical attributes of the finished tablets were maintained under the tested conditions; despite a pronounced post-storage matrix hardening effect, dissolution at the 45 min checkpoint and assay values remained within acceptance limits. Related-substance analysis confirmed the absence of meaningful chemical degradation, with 1-cyanoguanidine remaining well within acceptance limits throughout.

Collectively, these findings identify favorable pilot-scale processing conditions for granule micromeritics, tableting performance, and film-coated tablet quality attributes of this exceptionally high-load metformin hydrochloride formulation; extrapolation to industrial-scale manufacturing and formal process robustness will require further evaluation using multivariate/design-of-experiments approaches and scale-dependent process validation. Beyond their immediate manufacturing relevance, these findings highlight that parameter interactions—rather than single factors in isolation—should be considered when developing high-dose, poorly compressible oral solid dosage forms, a principle applicable beyond metformin to other actives with similar compressibility challenges. Clinically, maintaining consistent tablet hardness, dissolution, and stability under these conditions helps ensure reliable therapeutic dosing for patients dependent on high-dose metformin hydrochloride therapy.

## Figures and Tables

**Table 1:** The complete qualitative and quantitative composition of the metformin hydrochloride core tablet and the coating dispersion.

**Component**	**Function**	**% *w*/*w* of Core Tablet**	**Amount/Tablet (mg)**
Metformin hydrochloride	Active ingredient	93.633	1000
Polyvinylpyrrolidone K30	Binder	3.745	40
Sodium starch glycolate	Disintegrant	0.936	10
Colloidal silicon dioxide	Glidant	0.749	8
Magnesium stearate	Lubricant	0.936	10
Total core weight		100.000	1068
**Component**	**Function**	**% *w*/*w* of Coating Solids**	**Amount/Tablet (mg)**
Hydroxypropyl methylcellulose 6 mPa·s	Film former	45.0	9
Macrogol 6000	Plasticizer	30.0	6
Titanium dioxide	Opacifier	15.0	3
Talc	Anti-tacking agent	10.0	2
Total nominal coating solids		100.0	20

**Table 2:** Manufacturing process parameters investigated and corresponding quality attributes.

Manufacturing Stage	Investigated Parameter	Evaluated Response
Wet granulation	Chopper speed	Compressibility index
	Mixing time	
Compression	Compression force	Appearance, hardness, dissolution
	Tableting speed	Appearance, hardness
Film coating	Pre-coating drying	Appearance, dissolution
	Coating temperature	Appearance, dissolution

**Table 3:** Effects of chopper speed and wet mixing time on granule physical properties.

Chopper Speed (rpm)	Wet Mixing Time (min)	Bulk Density (g/mL), (*n* = 3)	Tapped Density (g/mL), (*n* = 3)	Compressibility Index (%), (*n* = 3)
1400	3	0.467 ± 0.004	0.572 ± 0.005	18.29 ± 0.998
1400	5	0.482 ± 0.003	0.577 ± 0.006	16.51 ± 1.111
1400	7	0.543 ± 0.006	0.616 ± 0.014	11.88 ± 1.815
2800	3	0.487 ± 0.004	0.589 ± 0.002	17.31 ± 0.959
2800	5	0.419 ± 0.004	0.517 ± 0.004	18.89 ± 0.891
2800	7	0.480 ± 0.004	0.584 ± 0.009	17.80 ± 0.531

**Table 4:** Particle size distribution, moisture content, and granulation process yield of intermediate granules under the tested chopper speed and wet massing time conditions.

Batch	Chopper Speed (rpm)	Wet Massing Time (min)	D_50_ (µm) (Mean ± SD, *n* = 2)	Fines < 106 µm (%)	LOD (%) (Mean ± SD, *n* = 3)	Granulation Yield (%)
1	1400	3	257.75 ± 14.36	9.76	1.85 ± 0.03	96.00
2	1400	5	277.96 ± 25.78	6.89	1.87 ± 0.05	96.65
3	1400	7	508.94 ± 37.34	4.56	1.98 ± 0.13	95.55
4	2800	3	416.92 ± 134.09	7.35	1.99 ± 0.02	96.85
5	2800	5	392.73 ± 95.81	11.22	1.91 ± 0.10	95.70
6	2800	7	495.45 ± 52.98	7.16	2.03 ± 0.08	96.60

**Table 5:** Effects of compression force and tableting speed on tablet appearance.

Tableting Speed (rpm)	Compression Force (kN)	Tablet Appearance	
8	10	Acceptable tablet appearance	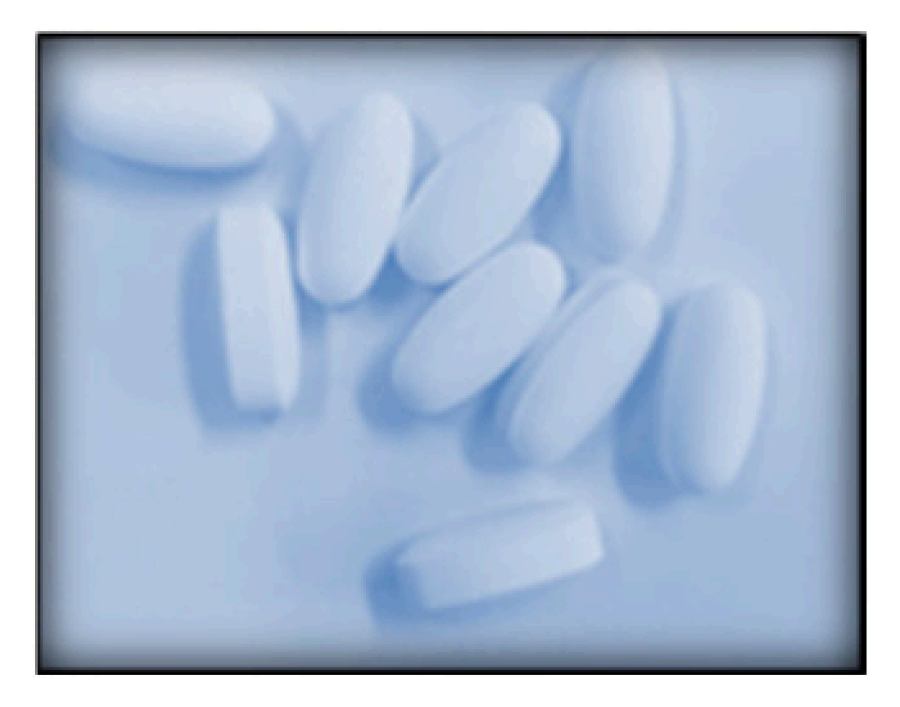
15			
20			
30			
40			
8	20		
15			
20			
≥30	20	Tablet capping observed	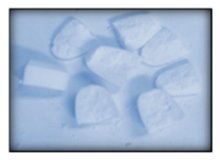
≥20	≥30		
≤15	≥30	Unstable machine operation	

**Table 6:** Effects of compression force and tableting speed on tablet hardness, thickness.

No.	Tableting Speed (rpm)	Compression Force (kN)	Tablet Hardness (kP), Mean ± SD (*n* = 10)		Tablet Thickness (mm), Mean ± SD (*n* = 10)	
			1st Batch	2nd Batch	1st Batch	2nd Batch
A1	8	10	6.31 ± 0.16	6.18 ± 0.20	7.90 ± 0.01	7.59 ± 0.04
A2	15	10	6.25 ± 0.14	6.29 ± 0.35	7.82 ± 0.01	7.81 ± 0.02
A3	20	10	6.48 ± 0.30	6.50 ± 1.64	7.80 ± 0.01	7.81 ± 0.18
A4	30	10	6.85 ± 0.36	7.31 ± 0.71	7.76 ± 0.01	7.45 ± 0.03
A5	40	10	7.37 ± 0.40	7.06 ± 0.70	7.76 ± 0.01	7.42 ± 0.02
A6	8	20	9.13 ± 0.60	8.14 ± 1.20	7.58 ± 0.01	7.49 ± 0.03
A7	15	20	9.07 ± 0.60	8.19 ± 2.35	7.48 ± 0.03	7.53 ± 0.09
A8	20	20	9.32 ± 0.55	7.52 ± 0.49	7.45 ± 0.02	7.43 ± 0.02
A9 *	30	20	3.38 ± 0.33	3.92 ± 1.13	7.90 ± 0.04	7.62 ± 0.03
A10 *	40	20	3.91 ± 1.11	3.20 ± 1.14	7.84 ± 0.25	8.38 ± 0.19

* Tablets at this condition exhibited mild capping but remained sufficiently intact for hardness testing; the low recorded values reflect incipient structural weakening rather than a fully intact compact.

**Table 7:** General linear model analysis of the effects of compression force and tableting speed on tablet hardness.

Source of Variation	SS	df	MS	F	*p*-Value	F Crit	Partial η^2^
Tablet-press speed	20.63508	4	5.15877	16.86755	0.000192	3.47805	0.871
Main compression force	0.03362	1	0.03362	0.109927	0.747071	4.964603	0.011
Press speed × compression force	40.13348	4	10.03337	32.80594	1.01 × 10^−5^	3.47805	0.929
Residual error (between-lot variation)	3.0584	10	0.30584				
Total	63.86058	19					

R^2^ = 0.952; Adjusted R^2^ = 0.909.

**Table 8:** Dissolution profile of core tablets (A1–A8) and a coated reference product, with corresponding f2 similarity factors.

No.	5 min (%)	10 min (%)	15 min (%)	45 min (%)	Sum (Rt − Tt)^2^	f2
A1	35.99 ± 2.77	76.11 ± 1.26	81.61 ± 0.86	99.53 ± 1.13	86.54	66.13
A2	30.30 ± 0.93	71.83 ± 0.63	78.67 ± 0.73	97.16 ± 1.52	31.81	76.20
A3	34.61 ± 0.73	75.67 ± 0.73	79.67 ± 0.62	98.11 ± 0.70	68.03	68.61
A4	35.24 ± 1.88	75.75 ± 1.14	81.00 ± 1.21	97.22 ± 0.84	69.57	68.38
A5	45.32 ± 2.27	78.60 ± 2.13	86.00 ± 0.87	100.38 ± 0.83	301.83	52.91
A6	34.40 ± 1.02	75.95 ± 0.86	83.22 ± 0.86	101.55 ± 0.76	100.19	64.61
A7	32.02 ± 3.13	74.29 ± 0.45	79.95 ± 0.46	98.43 ± 0.62	49.82	71.78
A8	31.17 ± 2.06	73.55 ± 0.59	77.30 ± 1.39	98.75 ± 0.89	54.13	70.94
Reference	33.62 ± 4.33	67.60 ± 2.81	80.35 ± 2.53	96.90 ± 1.11	—	—

A1–A8: core (uncoated) tablets, *n* = 6. Reference: coated reference (innovator) product. Dissolution profile and f2 determined in the primary dissolution medium (phosphate buffer, pH 6.8) only.

**Table 9:** Disintegration time and friability of core tablets under the tested compression conditions.

No.	Speed (rpm)	Force (kN)	Disintegration Time (min, *n* = 6, 1st Batch)	Friability (%, *n* = 10, 1st Batch)
A1	8	10	21.25–22.65	0.59
A2	15	10	21.33–22.92	0.54
A3	20	10	24.12–26.08	0.57
A4	30	10	24.42–26.05	0.52
A5	40	10	23.75–25.00	0.49
A6	8	20	21.25–24.05	0.44
A7	15	20	22.08–24.07	0.46
A8	20	20	23.17–24.62	0.41

**Table 10:** Effects of pre-coating drying conditions and coating temperature on tablet appearance and dissolution performance.

No.	Pre-Coating Drying Condition	Tablet Bed Surface Temperature (°C)	Tablet Appearance	Dissolution (%)			
				5 min	10 min	15 min	45 min
F1	With drying	30	Acceptable	45.68 ± 3.67	80.07 ± 0.97	97.83 ± 0.28	102.40 ± 1.56
F2	With drying	35	Acceptable	47.88 ± 2.65	87.90 ± 2.13	101.25 ± 0.13	98.81 ± 0.92
F3	With drying	40	Acceptable	48.35 ± 0.86	79.91 ± 2.43	98.30 ± 2.31	100.89 ± 1.56
F4	With drying	45	Acceptable	47.96 ± 0.92	79.22 ± 1.46	92.09 ± 2.31	101.35 ± 0.41
F5	With drying	50	Acceptable	52.21 ± 0.77	80.80 ± 3.77	94.90 ± 0.30	99.33 ± 0.55
F6	Without drying	30	Acceptable	43.73 ± 2.69	78.76 ± 3.24	95.66 ± 2.80	101.40 ± 0.51
F7	Without drying	35	Acceptable	41.02 ± 0.64	78.12 ± 1.60	95.18 ± 0.81	99.84 ± 0.28
F8	Without drying	40	Acceptable	45.65 ± 0.10	85.78 ± 2.42	103.33 ± 1.48	99.22 ± 1.11
F9	Without drying	45	Acceptable	42.35 ± 0.95	77.59 ± 2.14	99.83 ± 0.74	100.81 ± 0.83
F10	Without drying	50	Acceptable	43.04 ± 1.37	80.00 ± 2.65	101.16 ± 0.96	100.16 ± 0.80

**Table 11:** Summary of two-factor ANOVA results for the dissolution of film-coated tablets.

Time (min)	With Drying, Mean (%)	Without Drying, Mean (%)	Bed Temperature, F (*p*)	Pre-Coating Drying, F (*p*)	Interpretation
5	48.416	43.158	0.925 (0.529)	15.584 (0.017)	A nominal difference was observed, but it may reflect early-time dissolution-test variability and was not considered conclusive
10	81.58	80.05	0.547 (0.713)	0.379 (0.571)	No statistically significant effect of either factor was detected
15	96.874	99.032	0.384 (0.812)	0.653 (0.464)	No statistically significant effect of either factor was detected
45	100.556	100.286	3.23 (0.141)	0.267 (0.633)	No statistically significant effect of either factor was detected

(*p*): Values presented as F-statistic (*p*-value).

**Table 12:** Effects of thermal-stress conditions on physical properties, dissolution, and assay of metformin hydrochloride tablets.

Study Batch	Stress Condition	Duration	Hardness (kP)	Weight (mg)	Dissolution (%)	Assay (%)	1-Cyanoguanidine (%)
Reference	30 °C/75% RH	Initial	23.73 ± 3.76	1072.52	96.90 ± 1.11	99.92 ± 0.24	0.002
	65 °C/75% RH	1 week	32.24 ± 3.13	1070.23	96.64 ± 1.84	97.26 ± 1.25	0.002
	65 °C/75% RH	2 weeks	32.30 ± 2.08	1065.31	96.81 ± 1.39	99.23 ± 0.07	0.003
Test	30 °C/75% RH	Initial	16.81 ± 1.31	1092.14	101.35 ± 0.41	98.22 ± 0.11	0.006
	40 °C/75% RH	1 week	26.67 ± 1.16	1095.50	98.45 ± 1.70	97.52 ± 0.24	0.005
	50 °C/75% RH	1 week	26.74 ± 3.03	1095.66	88.90 ± 6.36	97.76 ± 0.17	0.006
	65 °C/75% RH	1 week	31.97 ± 2.08	1090.57	90.65 ± 5.97	98.15 ± 0.07	0.005
	40 °C/75% RH	2 weeks	24.73 ± 1.38	1095.21	97.25 ± 1.12	99.04 ± 0.24	0.006
	50 °C/75% RH	2 weeks	28.53 ± 1.96	1094.49	97.07 ± 0.97	99.79 ± 0.07	0.005
	65 °C/75% RH	2 weeks	28.55 ± 1.22	1089.06	98.33 ± 1.62	98.59 ± 0.32	0.005

**Table 13:** Summary of key manufacturing process parameter–quality attribute relationships identified in this study.

Manufacturing Stage	Parameter(s)	Quality Attribute Affected	Key Finding	Section
Wet granulation	Chopper speed/wet massing time	Compressibility Index (*CI*)	Minimum *CI* (11.88%) at 1400 rpm/7 min; no consistent trend at 2800 rpm	Section 3.1.1
		Particle size (D_50_), fines fraction	D_50_ increases and fines decrease with massing time at 1400 rpm; irregular, more variable at 2800 rpm	Section 3.1.1
Compression	Compression force × tableting speed	Tablet hardness	Dominant force × speed interaction (partial η^2^ = 0.929); high force + high speed → mild capping	Section 3.2.2
		Tablet thickness	Anomalous thickness increase at 20 kN/≥30 rpm corroborates capping mechanism	Section 3.2.2
		Dissolution (45 min)	Insensitive to force/speed (97.2–101.6%); substantiated by disintegration data	Section 3.2.3
		Disintegration time/friability	Narrow range (21.25–26.08 min); friability ≤ 0.59%	Section 3.2.3
Film coating	Pre-coating drying/bed temperature	Tablet appearance	No visible coating defects across 30–50 °C	Section 3.3.1
		Dissolution profile	No significant effect at 10/15/45 min; minor, non-persistent effect at 5 min only	Section 3.3.2
Stability	Thermal stress (40–65 °C, 1–2 weeks)	Hardness, dissolution, assay, related substances	Matrix hardening observed, but dissolution, assay, and 1-cyanoguanidine remained within acceptance limits	Section 3.4.1, Section 3.4.2 and Section 3.4.3

## Data Availability

The data presented in this study are available on request from the corresponding author.

## Abbreviations

The following abbreviations are used in this manuscript:

CI	Carr’s compressibility index
GLM	General Linear Model
SD	Standard deviation
API	Active pharmaceutical ingredient
RH	Relative humidity
USP	United States Pharmacopeia
EP	European Pharmacopoeia
